# Authentication of “Adelita” Raspberry Cultivar Based on Physical Properties, Antioxidant Activity and Volatile Profile

**DOI:** 10.3390/antiox9070593

**Published:** 2020-07-06

**Authors:** Arantzazu Valdés García, Salvador E. Maestre Pérez, Mikita Butsko, María Soledad Prats Moya, Ana Beltrán Sanahuja

**Affiliations:** Analytical Chemistry, Nutrition and Food Science Department, University of Alicante, P.O. Box 99, E-03080 Alicante, Spain; salvador.maestre@ua.es (S.E.M.P.); butsko.m.s@gmail.com (M.B.); maria.prats@ua.es (M.S.P.M.); ana.beltran@ua.es (A.B.S.)

**Keywords:** authentication, raspberry cultivar, polyphenols, experimental design, volatile composition, antioxidant activity

## Abstract

Agricultural selection programmes are, today, working hard to obtain novel raspberry cultivars with higher nutritional and commercial quality. One of those cultivars is “Adelita”. This study aims to provide novel tools for raspberry cultivar identification—more specifically, the differentiation of “Adelita” from other raspberry cultivars. To perform this study, five “Adelita” samples were analysed—four cultivated in Spain and one, in Morocco—and they were compared to seven samples from six raspberry cultivars (“P04”, “Lupita”, “Enrosadira”, “P10”, “Quanza” and “Versalles”). The physical parameters (mass, length, equatorial diameter and firmness) combined with the Total Phenolic Content (TPC); the antioxidant capacity according to the antioxidant activity tested with the 2,2-diphenyl-1-picrylhydrazyl (DPPH), ferric-reducing antioxidant power (FRAP) and 2,2-azinobis (3-ethylbenzothiazoline-6-sulfonic acid) diammonium salt (ABTS) methods; and the main target volatile compounds were used as independent variables. Principal component and cluster analysis showed that the target volatiles and physical parameters together with the TPC and DPPH values could be useful to classify Adelita cultivars separately from the rest of the cultivars included in the work. Those results proved that the developed methodology could be proposed as a reliable approach for the identification of cultivar fraud in the supply chain.

## 1. Introduction

Red raspberries (*Rubus idaeus L.*, family: *Rosaceae*) are fruits appreciated by consumers for their sharp colour, delicate texture, unique flavour and nutritional value. The worldwide production of raspberries increased by 67% from 2010 to 2018. Russia is the highest raspberry producer (19%), followed by Mexico (14.9%), Serbia (14.6%), Poland (13%), the United States (11%), Spain (5%) and Ukraine (4%) [1].

The external appearance and texture are critical quality attributes of raspberries, especially their firmness, due to being associated with freshness and the fruit’s resistance to damage during the harvesting, distribution and marketing processes [2]. Thus, raspberry cultivars that produce large, shiny and firm fruits are of high interest in the food industry. Regarding their nutritional value, raspberries are low in calories and an essential source of antioxidant compounds, in particular, polyphenols of the subgroup of flavonoids, which are renowned for their health benefits [3].

The aroma profile of raspberries—which is a complex combination of aldehydes, ketones, terpenes, alcohols, esters and furans [4]—has a significant impact on the consumer acceptability of fruits. Headspace Solid-Phase Microextraction in combination with Gas Chromatography-Mass-Spectrometry (HS-SPME-GC-MS) has been used for the volatile profiling of different raspberry cultivars. This technique is sensitive, is solvent-free in the extraction step, requires minimal sample preparation, and can be automated [4,5]. However, the extraction of volatiles is a complex process affected by different variables (i.e., the extraction temperature and time or sample weight), so the use of a statistical technique, such as response surface methodology (RSM), is often required to optimise the extraction conditions. Recently, RSM was successfully applied to optimise the extraction process for volatile compounds in blackberry fruits [6], but its specific use for volatile extraction in raspberry has not been reported yet.

Despite its benefits, the raspberry is a delicate and highly perishable fruit. Over-ripening, excessive softening and pathogen attack, mainly by the necrotroph *Botrytis cinerea*, are the leading causes of raspberry fruit postharvest losses [7]. Thus, R&D programmes try to develop new cultivars with higher-quality fruits that will lead to profitability for the farmer and to an increase in the demand from the consumer [8]. In particular, the novel “Adelita” cultivar was obtained as a result of a patented invention. Consequently, “Adelita” was selected due to being a cultivar available all year, with an abundant production of attractive large red-coloured fruits, with a uniform conical shape, very long shelf life and slightly acidic but sweet flavour [8]. These characteristics mean that the “Adelita” cultivar is constantly gaining market share on all continents.

The aim of the present work was the development of a low-cost and non-laborious procedure for differentiating “Adelita” from other raspberry cultivars. To this end, morphological data—such as the fruit length, equatorial diameter, fruit mass and firmness—were determined. Besides, the TPC; the antioxidant capacity as determined with the ferric-reducing antioxidant power (FRAP), 2,2-diphenyl-1-picrylhydrazyl (DPPH) and 2,2-azinobis (3-ethylbenzothiazoline-6-sulfonic acid) diammonium salt (ABTS) methods; and the main target volatile compounds obtained by HS-SPME-GC-MS were also included. Finally, multivariate data analysis was used for the optimisation of the volatile extraction process and to detect differences between samples.

## 2. Materials and Methods

### 2.1. Reagents

Sodium carbonate, sodium chloride, glacial acetic acid, ferric chloride and potassium persulfate of analytical grade; methanol (HPLC grade); and n-hexane (99%, GC grade) were obtained from Panreac (Barcelona, Spain). Gallic acid monohydrate, (±)-6-hydroxy-2,5,7,8-tetramethylchromane-2-carboxylic acid (Trolox), Folin and Ciocalteu’s phenol reagent (2M), 2,2-diphenyl-1-picrylhydrazyl (DPPH), 2,4,6-tris(2-pyridyl)-s-triazine (TPTZ), 2,2-azinobis (3-ethylbenzothiazoline-6-sulfonic acid) diammonium salt (ABTS), hexanal, decanal, nonanal, linalool, α-ionone and β-ionone were acquired from Sigma-Aldrich Inc. (St. Louis, MO, USA).

### 2.2. Samples

Twelve different raspberry samples from Soloberry S.L. and local markets coded from S1 to S12 were included in the study. The cultivars “P04” (S2), “Lupita” (S3), “Enrosadira” (S4), “P10” (S5), “Quanza” (S6), “Versalles” (S8) and “Adelita” (S9, S10, S11 and S12) were all from Huelva (Spain), whereas “Adelita” (S1) and “Lupita” (S7) were from Kenitra (Morocco). The Huelva cultivars were harvested at a latitude of 37,266°, longitude of −6940° and 5 m altitude, whereas the samples obtained from Kenitra were harvested at a latitude of 34,261°, longitude of −6580° and 19 m altitude. Both regions are characterised by moderate climates. The average temperature during the growing seasons was 17 ± 5 °C. The precipitation probability in the growing seasons was 18 ± 3%, and the humidity levels were below 10%.

All the samples were manually harvested in the same period of May at the light red-ripe stage of maturity, visually classified according to the NCS—Natural Colour System^®©^ (colour number 2) [9], as shown in the Supplementary data (Figure S1). In the laboratory, 150 g of each raspberry cultivar was placed onto transparent polyethylene terephthalate (PET) trays, with small circular holes in the lids to improve the circulation of gases, and with a water absorption single-layer film inside. Damaged fruits were removed before analysis.

### 2.3. Physical Measurements of Samples

After the sample’s reception, three different individuals from each sample were analysed to obtain triplicates of each physical measurement. Firstly, the mass of each fruit was acquired with a precision of 0.001 g. Then, the length and equatorial diameter, measured at the medium third of the fruit, were obtained for each sample. The firmness of the individual fruits was measured with a handheld electronic PCE-FM 200 dynamometer (PCE-Ibérica, Albacete, Spain) for compression measurements lasting 3 s, with an accuracy of ±0.5% of the load, using a plunger with a 10 mm diameter following the instructions for similar fruits such as strawberries [2]. This test measures the force needed to press the plunger about 2 mm vertically downwards into the fruit. Afterwards, the samples were stored under vacuum in PET bags and frozen at −18 °C until being further analysed. Before the analysis, the berries were defrosted at room temperature.

### 2.4. Analysis of Volatile Compounds by HS-SPME-GC-MS

#### 2.4.1. HS-SPME-GC-MS Procedure

The required amount of sample was weighed in a 20 mL amber vial. Then, 1 mL of deionised water and a polytetrafluoroethylene (PTFE) stirring rod were incorporated. The vial was then sealed with an aluminium crimp cap provided with a needle-pierceable polytetrafluoroethylene/silicone septum. The SPME fibre used was divinylbenzene/carboxen/polydimethylsiloxane (DVB/CAR/PDMS) 50/30 mm, StableFlex, 1 cm long, mounted to an SPME manual holder assembly (Supelco, Bellefonte, PA, USA). In the literature, this fibre was reported as suitable for the extraction of volatile compounds from raspberries [4]. The sample vial was placed in a water bath under temperature control and at 500 rpm stirring speed for 10 min for sample equilibration. The SPME needle was inserted into the vial through the septum, and the fibre was exposed to the vial headspace. After the required extraction time, the fibre was immediately desorbed into the GC-MS injection port at 250 °C for 10 min (splitless mode) on an Agilent 6890N GC coupled to a 5973N MS (Agilent Technologies, Palo Alto, CA, USA) operating in electron ionisation mode (EI 70 eV). The ion source and GC-MS transfer line temperatures were 230 and 280 °C, respectively. A DB-624 column, 30 m × 0.25 mm × 0.14 mm (Agilent Technologies, Palo Alto, CA, USA), was used, and it was programmed to change from 50 (hold, 2 min) to 250 °C at a rate of 10 °C min^−1^ (hold 12 min). Helium was used as the carrier gas (1 mL min^−1^). Blank runs were carried out before sample analysis to verify a lack of contaminants on the fibre. Peak identification was based on the comparison of mass spectrum data with spectra in full scan mode (*m/z* 30–550) present in the Wiley library, considering the volatile compounds that had equal to or more than 90% similarity. In this study, six raspberry volatile markers were selected: linalool, α-ionone, β-ionone, hexanal, decanal and nonanal. All of them were quantified using calibration curves at six concentration levels prepared in deionised water. All determinations were carried out in triplicate.

#### 2.4.2. Optimisation of HS-SPME Procedure

RSM was employed to assess the effects of the most relevant HS-SPME extraction variables, i.e., factors, on the signals of the selected volatile compounds of the raspberry samples. The effects of three independent factors (sample weight, extraction temperature and extraction time), at three levels each, on a dependent variable were studied. The dependent variable was the sum of the areas of the six volatiles selected. A Box–Behnken design (BBD) was used because this model had been successfully applied for the optimisation of the extraction, by HS-SPME-GC-MS, of volatile compounds in blackberry samples [6]. As shown in Table 1, a total of 16 experiments (3-level design including a 12 subset of the runs in the full three-level factorial and four centre points to estimate the experimental error) were carried out in a randomised order.

### 2.5. Preparation of Antioxidant Extracts

The extraction of antioxidants was carried out with a mixture of methanol/deionised water/HCl (1%) (70:29:1) according to a slightly modified methodology [10]. Raspberries were pureed in a ceramic mortar with a pestle. Subsequently, 1.0 ± 0.1 g of the raspberry mash was weighed in a polyethylene test tube, and 4 mL of the extraction mixture was added. The mixture was vortexed for 1 min and then left to stand for 16 h in the fridge. Afterwards, the tubes were vortexed again for 1 min and then centrifuged at 5000 rpm for 10 min. The supernatant was collected and passed to a new tube with a Pasteur pipette. The extraction process was repeated twice but without leaving the sample in contact with the extractant overnight. The combined extracts were stored in the freezer at −18 °C until analysis. Samples were extracted in triplicate.

### 2.6. Total Polyphenols Content (TPC)

The TPC assay was performed according to previous work with some modifications [11]. A volume of 200 µL of the methanolic extract was mixed with 100 µL of Folin and Ciocalteu’s phenol reagent (2 N) and 500 µL of a 7% sodium carbonate solution. The mixture was incubated at room temperature for 90 min. The absorbance was measured at 760 nm in a spectrophotometer (Biomate-3, Thermospectronic, Mobile, AL, USA) using deionised water as the blank. The results are expressed as mg gallic acid equivalents (GAE) per 100 g of sample. The TPC was determined for three different extracts of each sample.

### 2.7. Antioxidant Capacity

Three methods were used to determine the antioxidant activity of the extracts: the radical scavenging activity by DPPH method, the ABTS radical cation scavenging assay and the FRAP method. The existence of a collection of antioxidant capability determination assays can be understood when considering that, when the oxidation processes develop in vivo, different reactive species and several mechanisms are involved [12]. A combination of methods is convenient for characterising the antioxidant capacity of a sample [13]. All the tests were done in triplicate using a spectrophotometer (Biomate-3, Thermospectronic, Mobile, AL, USA). A standard curve was prepared using TROLOX as a standard in a range of concentrations from 0 to 500 µmol L^−1^. The results are expressed as µmol equivalents of TROLOX per 100 g of sample.

The DPPH method employed was a slight modification of one previously reported by Gramza-Michalowska et al. (2019) [14]. An aliquot of 50 µL of the raspberry extract was added to 3 mL of an ethanolic solution of DPPH^•^ (0.025 g L^−1^). The absorbance at 517 nm was registered every minute for 3 h to evaluate when the reaction reached a plateau, to ensure a stable value of the absorbance. For this application, the reaction time selected was 30 min. Subsequently, all the samples were measured spectrophotometrically after 30 min of incubation in the dark at room temperature (25 ± 2 °C).

The radical DPPH scavenging capacity (AA%) and the ABTS scavenging activity were determined according to Masci et al. (2016) [15]. The ABTS radical cations were prepared by mixing 25 mL of 7 mM ABTS solution with 88 µL of potassium persulfate (140 mM) solution. The solution obtained was kept in the dark for 16 h. The ABTS solution was conveniently diluted with 96% ethanol until an absorbance value of 0.80 ± 0.02 was obtained at 734 nm. Then, 50 µL volumes of the extracts (80–100 mg raspberry mL^−1^) were combined with 3 mL of the ABTS solution and vortexed. The reaction mixture was incubated at room temperature (25 ± 2 °C) for 30 min, and then the absorbance was measured at 734 nm against a blank (ABTS solution with 100 µL of methanol/water (80:20)).

The reductive capacity of the ferric cations of the methanolic extracts was assessed according to Benzie and Strain (1996) with several modifications [16]. The FRAP reagent was prepared freshly every day by mixing a sodium acetate buffer (300 mM, pH 3.6) with a 10 mM solution of 2,4,6-tripyridylo-S-triazine (TPTZ) and a 20 mM FeCl_3_·3H_2_O solution in a volumetric ratio of 10:1:1. An extract aliquot of 40 µL was mixed with 3 mL of FRAP reagent and incubated for 30 min in the dark at 25 ± 2 °C. Measurements were performed at 593 nm.

### 2.8. Statistical Analysis

The experimental conditions that maximised the response from the BBD were obtained from the fitted model using the StatGraphics Centurion XV software (Statistical Graphics Corporation, Rockville, MD, USA). A one-way analysis of variance (ANOVA) and differences between means were assessed based on confidence intervals using the Tukey test at a confidence level of 95% (*p* < 0.05). Cluster analysis was taken into consideration for the quality control of the “Adelita” raspberry cultivars from the rest, whereas Principal Component Analysis (PCA) was proposed to extract the vital information from a multivariate data table. Correlations among the data obtained by the ABTS, DPPH and FRAP assays and TPC results were also proposed. All the statistical analysis was carried out by using the SPSS software (Version 15.0, Chicago, IL, USA).

## 3. Results and Discussion

### 3.1. Physical Analysis of Raspberry Cultivars

The results of the morphological measurements, mass and firmness of the raspberry fruits are presented in Table 2.

The values show a certain degree of variability due to the combined effects of the cultivar, growing ecological conditions and state of maturity. This variability precludes the differentiation of one of the cultivars from the others using these parameters. However, samples of the “Adelita” cultivar, from Morocco (S1) and Spain (S9, S10, S11 and S12), show somehow higher mass and length than the rest of the studied cultivars. Correlation analysis of the data revealed that the equatorial diameter is positively correlated with the fruit mass and length (*p* < 0.05), while firmness is negatively correlated with mass, length and equatorial diameter (*p* < 0.05). It is interesting to note that the S7 sample has the highest firmness and the lowest mass, length and diameter of the studied samples. As a result, less fruit softening could take place during raspberry postharvest storage, which occurs mainly by the disassembly of the cell walls of parenchyma cells and the loss of cell adhesion because of middle lamellar dissolution [17].

### 3.2. Optimisation of HS-SPME Procedure by BBD

The extraction temperature and time and sample weight were the selected variables, i.e., factors, for optimisation, based on previous references on the HS-SPME fractionation of volatiles from different berries [6,18]. Regarding the sample weight, it has been reported that it influences the concentration of the volatile compounds in the headspace due to the ratio of sample weight to headspace volume [5]. Thus, it is interesting to consider this parameter to the avoid saturation of the fibre [19].

Some compounds have been widely recognised in the literature as typical of the aroma profile of ripened raspberry fruits. C13-norisoprenoids, such as α- and β-ionone, are the most relevant components of the red raspberry aroma with characteristic aromatic notes of flowers and herbs [20]. Linalool is particularly important terpene linked to the red raspberry aroma [21]. Otherwise, aldehydes such as hexanal, decanal and nonanal have been reported to be typical volatiles of ripened raspberry fruit [6,22]. Thus, the response evaluated was the sum of the signals (absolute areas) obtained from hexanal, decanal, nonanal, linalool, α-ionone and β-ionone [6].

**Figure 1:**
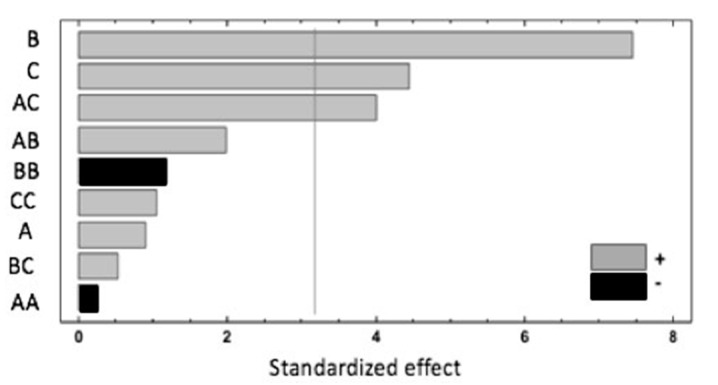
Pareto chart of factors and interactions obtained from the Box–Behnken design (BBD) for the response, where A = sample weight, B = extraction temperature and C = extraction time.

A summary of the results is shown in the Pareto chart (Figure 1). The extraction temperature (B) has the most significant influence on the response, showing a positive effect. Additionally, the extraction time (C) and the interaction of the sample weight and extraction time (AC) have a significant and positive effect. According to the ANOVA analysis, these three effects have *p*-values lower than 0.05, indicating that they are significantly different from zero at the 95.0% confidence level. The rest of the investigated parameters have no significant impact on the studied response.

These results could be explained by the effect of the temperature applied during the HS-SPME, which modified the raspberry cell walls, mainly due to pectin and hemicellulose depolymerisation that enhanced the extractability of the studied compounds [23]. Increasing the extraction temperature has been reported to be a good way of improving the extraction recovery, but high temperatures are also associated with the unwanted generation of artefacts such as BB interactions, which have been shown to have negative effects [5]. The significant and positive effects of the AC interaction underlined the fact that a higher sample weight and extraction time improves the recovery. However, the negative effect of the quadratic interaction of the sample weight (AA) could be related to the saturation of the closed vial headspace, increasing the competition between target and interfering compounds for absorption in the fibre coating and, consequently, their extraction. The following equation expresses the mathematical model representing the studied response as a function of the independent variables within the region under investigation:

Y = −4.22696E7 − 2.16458E8 × A + 1.06789E7 × B − 6.66752E6 × C − 6.18362E6 × A^2^ + 2.79469E6 × AB + 4.01389E6 × AC − 100127 × B^2^ + 32128.9 × BC + 45266.1 × C^2^

The R-squared statistics indicate that the model, as fitted, explains 88.8% of the variability in the response, demonstrating a good correlation between the actual and predicted values since R square is close to unity. Additionally, the lack-of-fit was not significant (F-value = 3.16) relative to the pure error, with a *p*-value higher than 0.05 (*p*-value = 0.1848). Using the model quoted above, the optimal HS-SPME conditions for obtaining the highest response of 2.82 × 10^8^ are sample weight = 1.36 g, extraction temperature = 60 °C and extraction time = 45 min. Triplicate HS-SPME-GC-MS determinations were carried out, obtaining a response of 2.68 × 10^8^ with a DER of 4.1% intra-day by performing three extractions under optimal conditions in a single day, and a DER of 7.2% inter-day based on three extractions under optimal conditions per day over three consecutive days (*n* = 9).

### 3.3. Validation of HS-SPME-GC-MS Method

The analytical method used for volatile quantification was validated in terms of its linearity, limits of detection (LOD) and quantitation (LOQ), and precision, considering the intra- and inter-day repeatability. Acceptable linearities were obtained using a set of calibration curves prepared with six standards. The LOQ and LOD values were determined by using regression parameters from the calibration curves at five concentration levels, in triplicate (3 S_a_/b and 10 S_a_/b, respectively, where S_a_ is the standard deviation of the residues and b is the slope). The repeatability is expressed as the relative standard deviation (RSD) of the peak areas of triplicates. The intra-day precision (*n* = 3) was estimated by performing three extractions under optimal conditions in a single day, and the inter-day precision (*n* = 9) was estimated based on three extractions performed under optimal conditions per day over three consecutive days. All the results are reported in Table S1 in the Supplementary Data.

### 3.4. Quantification of Target Volatile Compounds of Raspberry Cultivars

In this study, the selected cultivars had different quantitative compositions (Table 3). The terpene linalool is produced through the monoterpene breakdown pathway, and it is related to the floral and sweet sensory attributes of raspberries, whereas the C13 norisoprenoids α- and β-ionone are produced via lycopene breakdown, and they are responsible for the natural berry and violet sensory attributes of this fruit [24,25]. Since terpene and C13-nonisoprenoid compounds are linked with the characteristic floral and berry sensory attributes of raspberry fruits, the significantly higher content of the sum of the three most relevant components (β-ionone, α-ionone and linalool) of the S1, S9, S10, S11 and S12 samples could suggest that the “Adelita” cultivar had more floral aromatic notes [22].

In particular, the remarkably high amount of linalool found in the “Adelita” samples could be an advantage for this cultivar, since this terpene has been reported for its antifungal efficacy against *B. cinerea* in strawberries and blueberries [4,26]. Although further studies are necessary, these results could indicate that the quantification of this target compound in raspberries could be used as an indicator of resistance to *B. cinerea*.

Regarding the content of aldehydes, it has been reported that these compounds are notably produced from fatty acid breakdown occurring in the cell walls, being responsible for herbaceous and green odour notes [4,25]. The results obtained from this study underline that cultivar variation affects the volatile compound concentrations of raspberries. These results are in accordance with previous ones reported in the literature in which the characterisation of 14 raspberry cultivars was reported, suggesting a wide genetic variability [18].

Although interesting information was obtained about the target volatiles of the samples, no clear differences were observed between the raspberries corresponding to the “Adelita” cultivar and the samples belonging to the other evaluated cultivars. In particular, although taking into account the content of linalool alone seemed to facilitate the differentiation of the “Adelita” cultivar from the other samples, the variability for this parameter obtained by Tukey analysis inside the group of the “Adelita” cultivar underlined the necessity of carrying out a multidisciplinary statistical approach in this study.

### 3.5. Analysis of Total Polyphenol Content (TPC)

**Figure 2:**
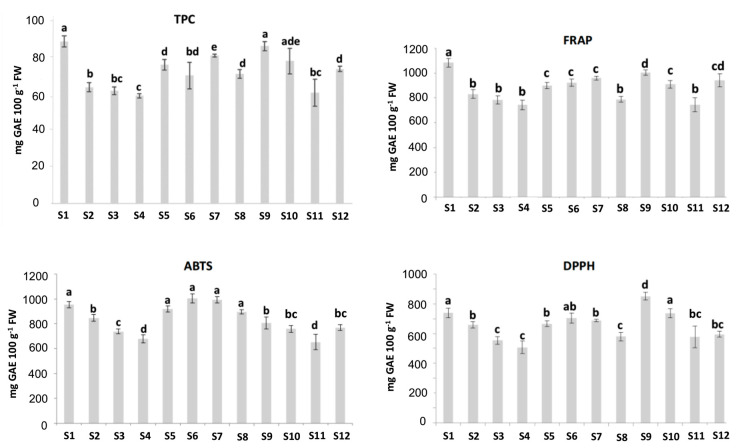
Total polyphenol content (TPC) (mg gallic acid equivalents (GAE) 100 g^−1^ FW), 2,2-azinobis (3-ethylbenzothiazoline-6-sulfonic acid) diammonium salt (ABTS), ferric-reducing antioxidant power (FRAP) and 2,2-diphenyl-1-picrylhydrazyl (DPPH) (µmol Trolox 100 g^−1^ FW) measurements for different raspberry cultivars from different origins; FW: Fresh weight. Results are expressed as mean ± standard deviation of three replicates for each sample (*n* = 3). Different letters (a,b,c,d,e) represent statistically significant differences (*p* < 0.05).

As shown in Figure 2, the TPC values varied greatly among the studied cultivars, ranging from 59.1± 1.3 to 88.8 ± 3.1 mg GAE 100 g^−1^ fresh weight (FW). The values found in the current study agreed with the ones reported in other studies [3,27,28]. In this study, higher TPC values were obtained for the “Adelita” samples S1 and S9, with values of 88.8 ± 3.1 and 86.3 ± 2.5 mg GAE 100 g^−1^ FW, respectively. On the other hand, the sample “Enrosadira” from Spain (S4) showed the lowest TPC value, which was 59.1 ± 1.3 mg GAE 100 g^−1^ FW. Concerning the TPC values and the contents of some polyphenols, Yang et al. (2020) [29] showed a relationship between the values of the TPC and the content of the polyphenols cyanidin-3-glucoside, catechin, epicatechin and proanthocyanidin B1, reported as the main phenols present in raspberries. Additionally, in the same study, ellagic acid, quercetin, kaempferol, gallic acid and caffeic acid were presented as noticeable phenolic compounds in raspberries. Regarding flavonols, quercetin-3-*O*-rutinoside, myricetin, luteolin and kaempferol were also confirmed by Ponder and Halmman (2019) [3], who pointed out raspberries as a rich source of polyphenolic compounds.

### 3.6. Antioxidant Capacity: DPPH, ABTS and FRAP Results

The antioxidant capacity of the samples was studied using the FRAP, ABTS and DPPH assays (Figure 2). The DPPH assay showed antioxidant capacities in the range of 507–850 μmol Trolox g^−1^ FW, lower than the values obtained for FRAP (743–1083 μmol Trolox g^−1^ FW) and ABTS (679–1003 μmol Trolox g^−1^ FW) analysis, as has been reported previously [30]. The DPPH and ABTS assays showed different antioxidant activities. The raspberry extracts had higher ABTS values relative to the DPPH scavenging activity, meaning there may be compounds in the samples that were responsible for the difference. Similar results were obtained by using these three methods in four tropical leafy vegetables [31]. In this sense, the ABTS assay measures the direct free radical inhibition by all the antioxidants in the raspberry extract (hydrophilic and lipophilic), whereas the DPPH assay is only applicable to hydrophobic systems [32,33].

The results of this work revealed a considerable variation in the antioxidant activity of the different raspberry cultivars. In the FRAP and DPPH assays, a similar trend was observed for all the samples, the antioxidant capacities being maximal in the “Adelita” samples (S1 and S9), which could be due to their higher TPC values as previously described. The main phenolic compounds of raspberries may block free radicals and prevent the reactions caused by a single active oxygen atom [14]. On the other hand, “Lupita”, “Enrosadira” and “P04” from Spain were always the cultivars with the lowest antioxidant capacity, their results being in line with the low values of TPC obtained for these samples previously reported in this work. Considering the TPC and antioxidant capacity values of the samples measured by FRAP, ABTS and DPPH methods, no clear differences were observed between the raspberries corresponding to the “Adelita” cultivar and the samples belonging to the other evaluated cultivars. Thus, a multidisciplinary statistical approach is proposed in this study.

### 3.7. Correlation between TPC and Antioxidant Methods

**Figure 3:**
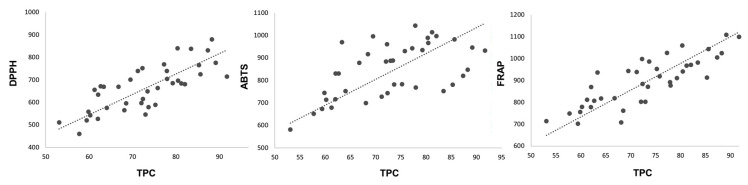
Correlations between radical scavenging capacity measured by DPPH, ABTS and FRAP methods (μmol Trolox 100 g^−1^ FW) and TPC (mg GAE 100 g^−1^ FW).

Positive correlations were found between the results of the three methods used to study the antioxidant capacity of the samples. The FRAP and DPPH results showed high significant correlation, with an *R*-value of 0.793, whereas the ABTS results showed a lower correlation with the FRAP and DPPH results, with R values of 0.524 and 0.620, respectively. Additionally, significant positive correlations (*p* < 0.05) were found between the DPPH, ABTS, and FRAP assay results with the TPC (Figure 3). The stronger correlation with the TPC suggests that the antioxidant activity of raspberries is derived mainly from the content of phenolic compounds, with significant and positive correlations (*p* < 0.05), especially between the FRAP results (*R* = 0.783) followed by the DPPH results (*R* = 0.734). The lowest correlation was found between the TPC assay and ABTS results (*R* = 0.576). This result may be, and is probably, related to the detailed structure of the specific compounds present in the samples. These results are in line with those previously reported in the literature [31], indicating that FRAP and DPPH are appropriate, little-time-consuming methods with high reproducibility for quickly determining antioxidant activity in raspberry fruit extracts.

The antioxidant properties of phenolic compounds are directly linked to their structure. As previously reported in the TPC section, the major phenols of raspberries—cyanidin-3-glucoside, catechin, epicatechin and proanthocyanidin B1—are composed of several aromatic rings bearing hydroxyl groups potentially able to quench free radicals by forming resonance-stabilised phenoxyl radicals [29,34].

### 3.8. “Adelita” Raspberry Cultivar Classification by Multivariate Analysis

**Figure 4:**
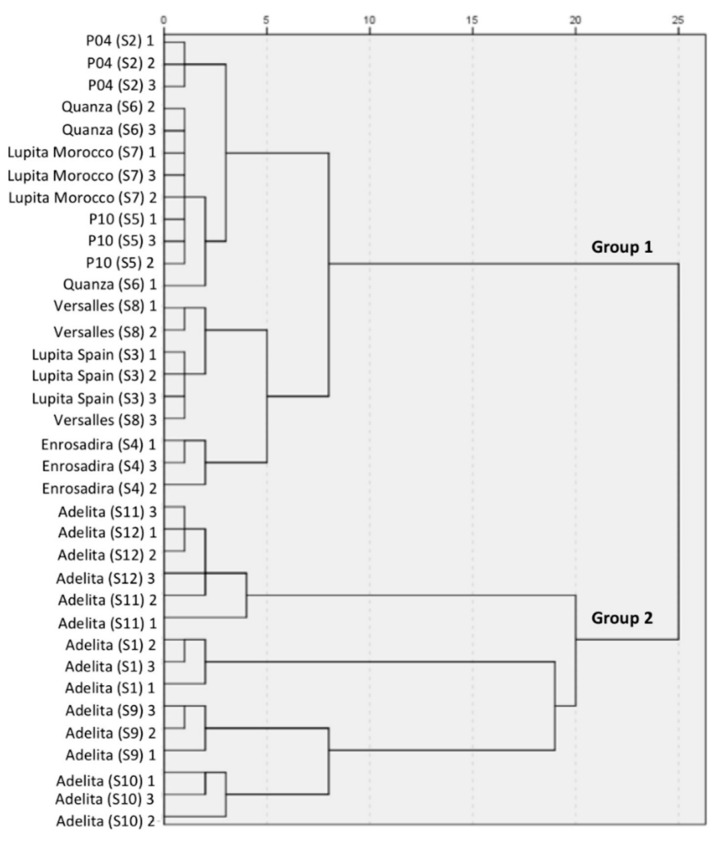
Hierarchical analysis dendrogram, obtained by cluster analysis method, of raspberry samples.

A hierarchical cluster analysis (HCA) to evaluate the similarity among raspberry cultivars was proposed in the present study. Sample similarities were assessed based on the squared Euclidean distance, and centroid clustering was used to group the samples. In this case, the data matrix was defined as 36 objects (three repetitions for twelve samples) and fourteen independent variables (mass; length; equatorial diameter; firmness; TPC; antioxidant activity according to the DPPH, ABTS and FRAP methods; and the content of the six studied target compounds (α- and β-ionone, linalool, hexanal, nonanal and decanal). Only twelve parameters allowed the differentiation of the “Adelita” cultivar and were included in the cluster analysis: mass, length, equatorial diameter, firmness, TPC, DPPH, α-ionone, β-ionone, linalool, hexanal, nonanal and decanal. As the dendrogram shows (Figure 4), similar groupings were obtained by which the 12 raspberry cultivars were clustered into two main groups when the 25-distance threshold was selected, suggesting that the “Adelita” cultivar could be clearly distinguished in Group 2 from the rest of the cultivars that were situated into Group 1. These parameters are very useful because if the cultivar of a raspberry sample is unknown, but information about the parameters included in the analysis is provided, it is possible to differentiate the “Adelita” raspberry cultivar from the other ones. Regarding the antioxidant capacity results, it is interesting to note that only the DPPH results were essential for the differentiation of the “Adelita” cultivar from the other ones. This fact could be related to the positive and significant correlation observed between the DPPH and TPC results. Although the FRAP results also showed similar positive and significant correlation with the TPC, their methodology requires the use of more chemicals and experimental time, so they were not included in the cluster analysis. Additionally, the absence of ABTS results in the HCA could be related to the lower significant correlation with the TPC and the results of the rest of studied antioxidant methods, as previously described in this work.

**Figure 5:**
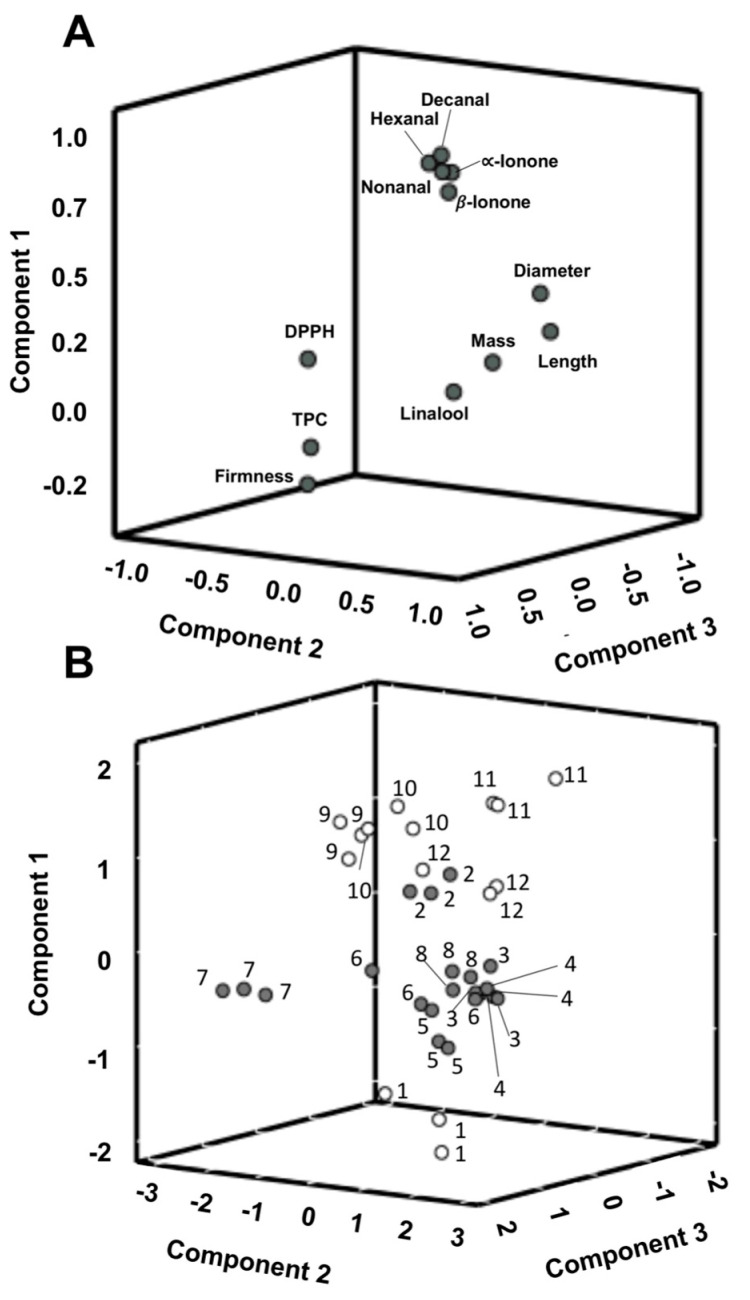
(**A**) 3-D projection of parameters onto a Principal Component Analysis (PCA) plot constructed from the principal component analysis of the data; (**B**) PCA-score plot of samples grouped in Group 1 (•) and Group 2 (°) of the cluster analysis with 1: sample “Adelita” (S1), 2: “P04” (S2), 3: “Lupita” (S3), 4: “Enrosadira” (S4), 5: “P10” (S5), 6: “Quanza” (S6), 7: “Lupita” (S7), 8: “Versalles” (S8), 9: “Adelita” (S9), 10: “Adelita” (S10), 11: “Adelita” (S11) and 12: “Adelita” (S12) and three field replications per raspberry cultivar.

The PCA applied to the twelve parameters included in the cluster analysis showed three principal components (PC) accounting for 76.8% of the total variation (48.5% PC1, 17.3% PC2 and 11.0% PC3). According to the 3D loading plot (Figure 5a), the main positive correlations with PC1 were for decanal, nonanal, hexanal, α and β-ionone, which were related to the intrinsic volatile profile and sensory properties of raspberries. PC2 could be related mainly to the physical properties of the samples since it exhibits positive loading mainly with mass, length and equatorial diameter and negative loading with firmness. This result corroborates the result obtained for S7 in the physical analysis section, which showed the highest firmness, although its size, length and diameter were the lowest of the studied samples. Additionally, linalool was positively loaded with this PC. Finally, PC3 primarily corresponds to positive loading of TPC and the DPPH results, and is related to the antioxidant activity of the raspberries.

According to the score plot (Figure 5b), all the studied samples were divided into two groups. The Group 2 obtained by the cluster analysis was composed of the “Adelita” cultivar samples (S1, S9, S10, S11 and S12) and the Group 1 was composed of the other cultivars (S2, S3, S4, S5, S6, S7 and S8). Thus, these results could suggest that, in general, the “Adelita” raspberry cultivar showed higher volatile contents of the target compounds related to floral aroma with high mass and broad and conical shape in contrast to the other studied cultivars.

## 4. Conclusions

The results obtained from this study proved that the cultivar could affect the chemical composition regarding the TPC and target volatile compounds, the antioxidant activity and physical the properties of the raspberries. Regarding the three antioxidant activity methods used, DPPH was the only method needed for the differentiation of “Adelita” from the rest of the cultivars combined with the other parameters included in the HCA. This result indicates an advantage since it is not a very tedious assay in terms of the preparation of the chemicals, and it is also operationally simple to perform compared with ABTS and FRAP. Our findings underlined the unique properties of the “Adelita” cultivar as a result of R&D efforts. The multidimensional statistical approach proposed with cluster analysis and PCA was interesting for detecting differences between samples, and it could help to visualise, with simplicity, the contribution of each parameter. This novel methodology could be proposed as a reliable and straightforward approach for the authentication of the “Adelita” cultivar to minimise the opportunity for food fraud in the supply chain. In order to validate the authentication process for the “Adelita” cultivar under more extensive agricultural and origin conditions, more samples should be introduced in a future study in which a Linear Discriminant Analysis could be used to establish the desired classification.

## Figures and Tables

**Table 1:** Box–Behnken experimental design showing the levels of each independent factor (sample weight, extraction temperature and extraction time) for the Headspace Solid-Phase Microextraction in combination with Gas Chromatography-Mass-Spectrometry (HS-SPME) extraction of target compounds in Raspberry S1.

Run	Sample Weight (g)	Extraction Temperature (°C)	Extraction Time (min)
1	2.0	60	27.5
2	1.25	60	45
3	0.5	47.5	10
4	1.25	35	45
5	1.25	35	10
6	1.25	47.5	27.5
7	2.0	47.5	10
8	1.25	47.5	27.5
9	0.5	35	27.5
10	1.25	60	10
11	2.0	35	27.5
12	0.5	60	27.5
13	1.25	47.5	27.5
14	1.25	47.5	27.5
15	2.0	47.5	45
16	0.5	47.5	45

**Table 2:** Results obtained for the mass, length, diameter and firmness of the studied raspberry cultivars expressed as average quantities ± standard deviations (*n* = 3).

Sample	Mass (g)	Length (cm)	Diameter (cm)	Firmness (N)
S1	7.08 ± 0.15 ^a^	2.8 ± 0.1 ^a^	2.7 ± 0.1 ^a^	4.1 ± 0.2 ^a^
S2	6.47 ± 0.08 ^b^	2.6 ± 0.1 ^ab^	2.6 ± 0.2 ^ab^	4.1 ± 0.3 ^a^
S3	5.31 ± 0.19 ^c^	2.5 ± 0.1 ^b^	2.7 ± 0.1 ^a^	3.5 ± 0.2 ^b^
S4	5.35 ± 0.17 ^c^	2.7 ± 0.1 ^b^	2.6 ± 0.2 ^ab^	4.6 ± 0.3 ^a^
S5	6.85 ± 0.10 ^b^	2.6 ± 0.1 ^b^	2.6 ± 0.1 ^b^	3.3 ± 0.1 ^c^
S6	6.53 ± 0.18 ^b^	2.5 ± 0.1 ^b^	2.6 ± 0.1 ^ab^	3.5 ± 0.1 ^bc^
S7	4.9 ± 0.3 ^d^	2.1 ± 0.2 ^c^	2.2 ± 0.1 ^c^	5.3 ± 0.2 ^d^
S8	5.45 ± 0.07 ^c^	2.5 ± 0.1 ^b^	2.9 ± 0.2 ^a^	4.0 ± 0.1 ^a^
S9	6.6 ± 0.4 ^ab^	2.7 ± 0.1 ^a^	2.8 ± 0.1 ^a^	3.5 ± 0.1 ^b^
S10	6.61 ± 0.19 ^ab^	2.7 ± 0.1 ^a^	2.8 ± 0.1 ^a^	3.4 ± 0.2 ^b^
S11	6.9 ± 0.2 ^ab^	2.8 ± 0.1 ^a^	2.9 ± 0.1 ^a^	3.5 ± 0.2 ^b^
S12	6.6 ± 0.3 ^ab^	2.8 ± 0.2 ^a^	2.8 ± 0.1 ^a^	3.7 ± 0.3 ^ab^

Different superscripts for each parameter (a,b,c) within the same column indicate statistically significantly different values (*p* < 0.05).

**Table 3:** Content of volatile compounds quantified by GC-MS expressed as mg per 100 g of raspberry fruit (mean ± SD, *n* = 3).

Sample	Hexanal	Linalool	Nonanal	Decanal	α-Ionone	β-Ionone
S1	0.8 ± 0.1 ^a^	260 ± 19 ^a^	56 ± 9 ^a^	8 ± 2 ^a^	41.5 ± 0.4 ^a^	212 ± 20 ^a^
S2	10.4 ± 1.2 ^b^	22 ± 2 ^b^	74 ± 4 ^b^	27 ± 6 ^b^	73 ± 5 ^b^	210 ± 5 ^a^
S3	5.7 ± 0.9 ^c^	16.85 ± 0.20 ^b^	48 ± 4 ^a^	6.3 ± 0.6 ^a^	40 ± 2 ^a^	134 ± 6 ^b^
S4	1.1 ± 0.3 ^a^	14.21 ± 0.10 ^b^	48 ± 5 ^a^	3.3 ± 0.8 ^c^	45 ± 6 ^a^	227 ± 26 ^ab^
S5	1.4 ± 0.5 ^a^	22.8 ± 0.8 ^b^	48 ± 3 ^a^	12 ± 2 ^a^	58.4 ± 0.3 ^c^	138 ± 19 ^b^
S6	4.6 ± 1.6 ^ce^	16.6 ± 1.7 ^b^	72 ± 3 ^b^	17 ± 2 ^d^	38.1 ± 1.5 ^d^	130 ± 11 ^b^
S7	1.4 ± 0.2 ^a^	42.9 ± 1.4 ^c^	44 ± 5 ^a^	4.6 ± 0.8 ^a^	39.8 ± 1.2 ^a^	141 ± 10 ^b^
S8	4.7 ± 0.7 ^c^	43 ± 4 ^c^	55 ± 5 ^ac^	22 ± 3 ^b^	42 ± 2 ^a^	140 ± 18 ^b^
S9	14 ± 4 ^bd^	86 ± 7 ^d^	102 ± 4 ^d^	57.8 ± 0.5 ^e^	66 ± 7 ^bc^	360 ± 17 ^c^
S10	15 ± 4 ^bd^	120 ± 11 ^de^	92 ± 4 ^d^	58.3 ± 1.2 ^e^	90 ± 8 ^be^	310 ± 22 ^c^
S11	16.5 ± 1.8 ^d^	154 ± 9 ^e^	97 ± 3 ^d^	61.7 ± 0.4 ^f^	92.8 ± 1.4 ^e^	360 ± 18 ^c^
S12	3.4 ± 0.4 ^e^	169 ± 13 ^e^	100 ± 6 ^d^	61.0 ± 0.8 ^ef^	90.4 ± 1.6 ^e^	351 ± 12 ^c^

Different superscripts (a,b,c,d,e,f) for each volatile compound within the same column indicate statistically significantly different values (*p* < 0.05).

## Supplementary Materials

The following are available online at https://www.mdpi.com/2076-3921/9/7/593/s1. Figure S1: Raspberry cultivars used in this study. Table S1: Validation parameters for the HS-SPME/GM-MS optimised method: Linear range (mg Kg^−1^), R^2^ value, LOD (μg Kg^−1^), LOQ (μg Kg^−1^), intra-day and inter-day repeatability (peak area RSD (%)).
