# Supplementary material for: Authentication of “Adelita” Raspberry Cultivar Based on Physical Properties, Antioxidant Activity and Volatile Profile
Source: Antioxidants (Basel). 2020 Jul 6;9(7):593. doi: 10.3390/antiox9070593 (PMC7402179; doi:10.3390/antiox9070593)
Supplement: Supplementary file 1 [file antioxidants-09-00593-s001.pdf]

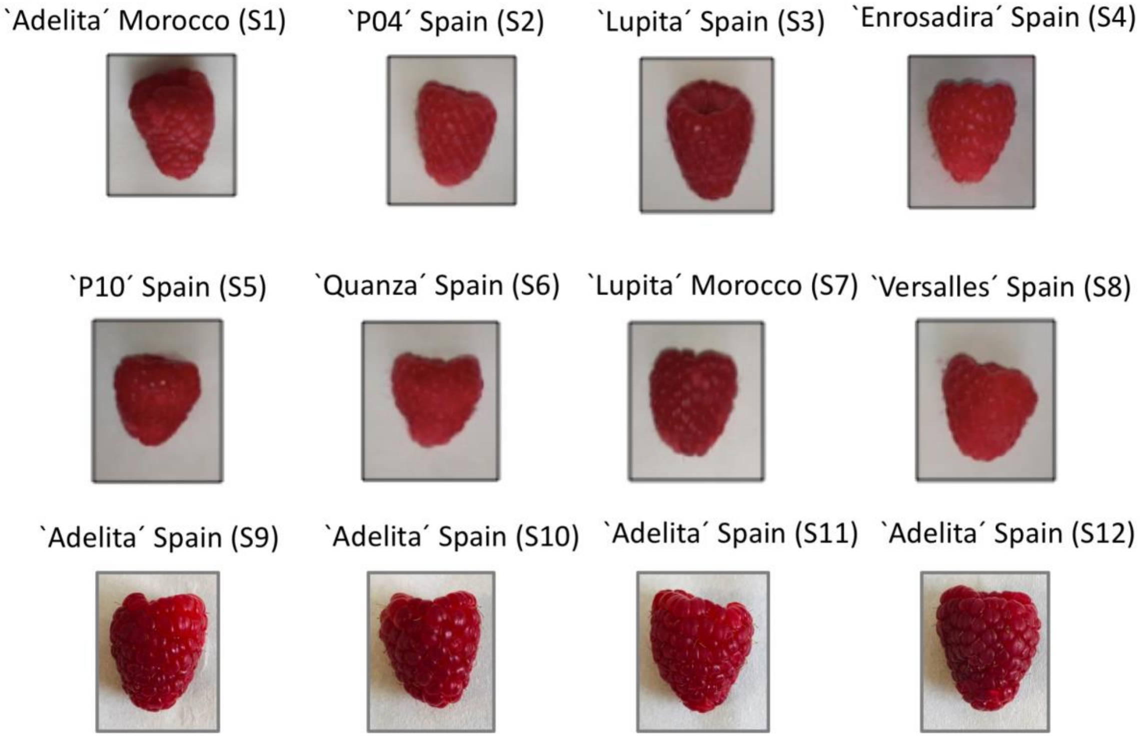

Figure S1. Raspberry cultivars used in this study.

Table S1. Validation parameters for the HS-SPME/GM-MS optimized method: Linear range (mg Kg^{-1}), R^2 value, LOD (µg Kg^{-1}), LOQ (µg Kg^{-1}), Intra-day and Inter-day repeatability (Peak area RSD (%)).

Parameters	Hexanal	Decanal	Nonanal	Linalool	α-Ionone	β-Ionone
Linear range	0.003-1.540	0.002-1.417	0.267-5.331	0.003-0.887	0.003-3.406	0.010-4.937
R^2 value	0.996	0.991	0.999	0.998	0.991	0.996
LOD	0.40 ± 0.04	0.44 ± 0.12	0.49 ± 0.03	0.58 ± 0.09	0.43 ± 0.03	0.46 ± 0.06
LOQ	1.33 ± 0.14	1.5 ± 0.4	1.63 ± 0.09	1.9 ± 0.3	1.42 ± 0.09	1.5 ± 0.2
Intra-day repeatability	1.5	0.9	10.7	7.6	0.7	2.3
Inter-day repeatability	7.3	10.5	12.4	12.1	4.3	6.6
